# Supplementary material for: Mechanism of Histone Arginine Methylation Dynamic Change in Cellular Stress
Source: Int J Mol Sci. 2024 Jul 10;25(14):7562. doi: 10.3390/ijms25147562 (PMC11277302; doi:10.3390/ijms25147562)
Supplement: Supplementary file 1 [file ijms-25-07562-s001.zip › ijms-3025710-supplementary.pdf]

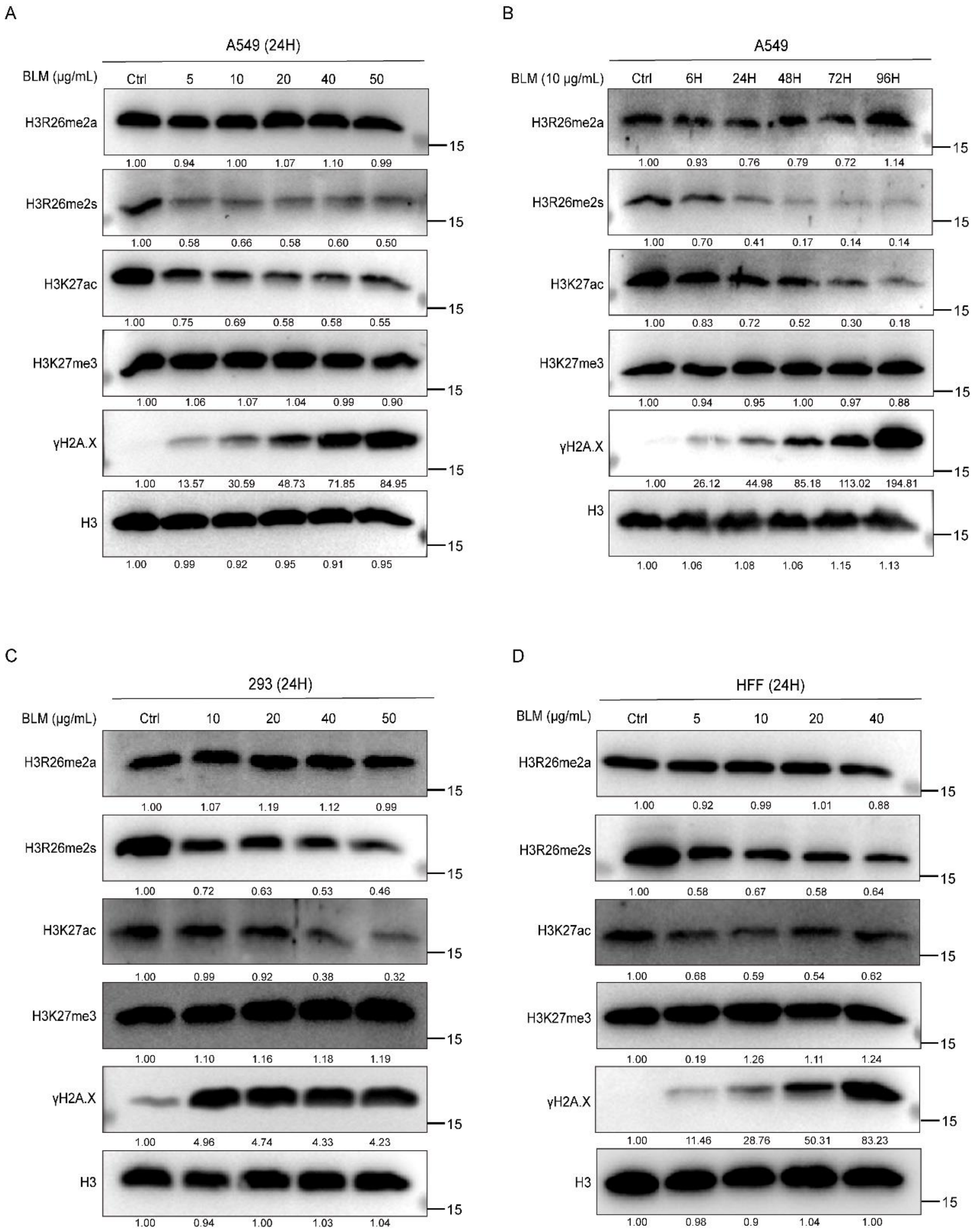

Figure S1. Histone H3R26me2s and H3K27ac modifications decrease were conserved in multi-cell lines.

(A) A549 cells were treated with 5 μg/mL, 10 μg/mL, 20 μg/mL, 40 μg/mL, and 50 μg/mL bleomycin for 24h, histone modifications were detected with the indicated antibodies. **(B)** A549 cells were treated with 10 μg/mL bleomycin for 6h, 24h, 48h, 72h, and 96h, and histone modifications were detected with the indicated antibodies. **(C)** 293 cells were treated with 10 μg/mL, 20 μg/mL, 40 μg/mL, and 50 μg/mL bleomycin for 24h, histone modifications were detected with the indicated antibodies. **(D)** HFF cells were treated with 10 μg/mL, 20 μg/mL, 40 μg/mL, and 50 μg/mL bleomycin for 24h, histone modifications

were detected with the indicated antibodies.

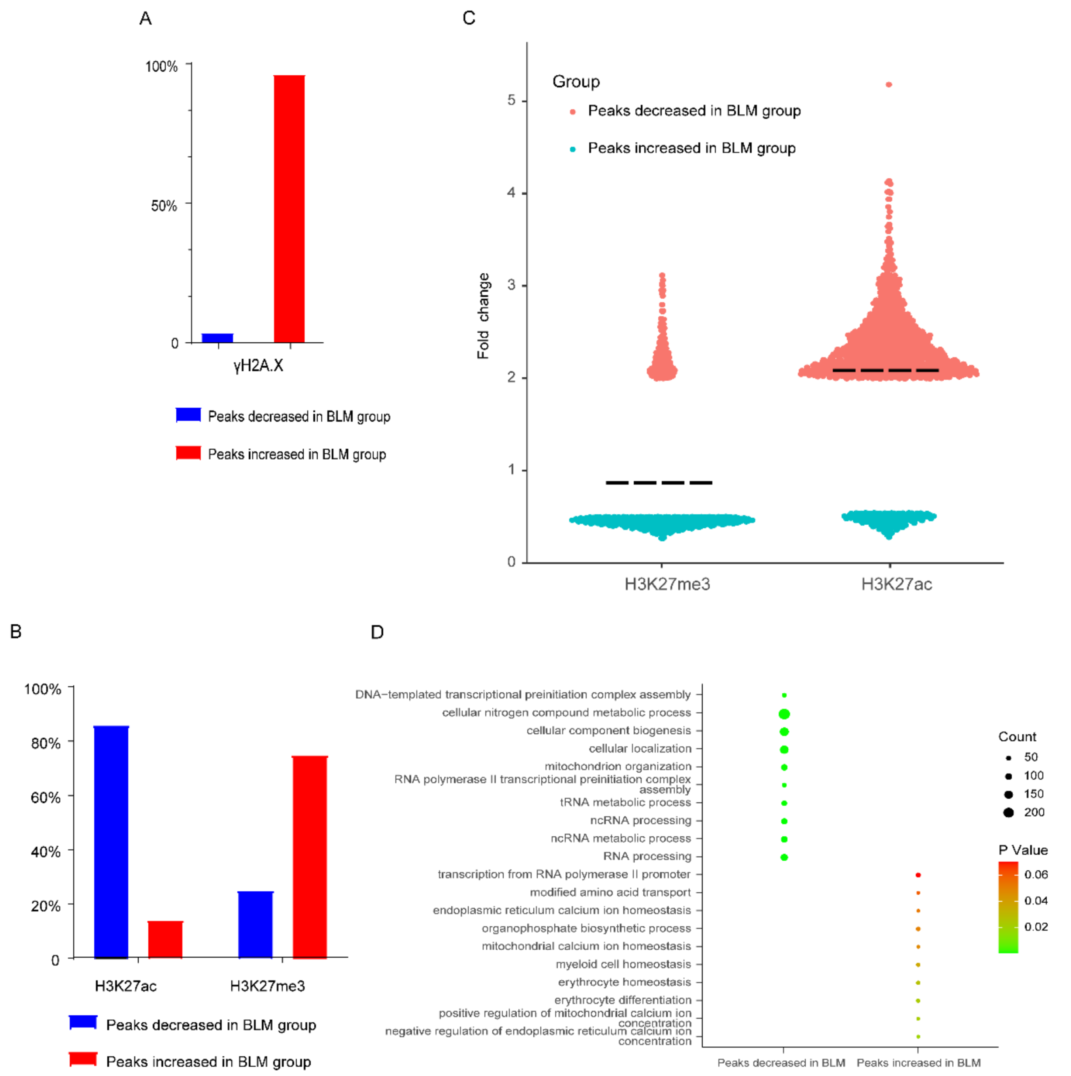

Figure S2. H3K27 modifications different peaks and associated gene analysis. **(A)** Percentage of γH2A.X up and down-regulated peaks in bleomycin treat cells. γH2A.X peaks were mainly upregulated. **(B)** Percentage of H3K27ac and H3K27me3 up and down-regulated peaks in bleomycin treat cells. H3K27ac peaks were mainly downregulated and H3K27me3 peaks were mainly up-regulated. **(C)** The number and fold change of histone modifications in different peaks were calculated, each dot represents a different peak, the black dashed line indicated the median of total peak change, and the median below 1 or above 1 represents histone modifications global changes. **(D)** Gene Oncology of H3K27ac sites modifications different peaks associated genes.

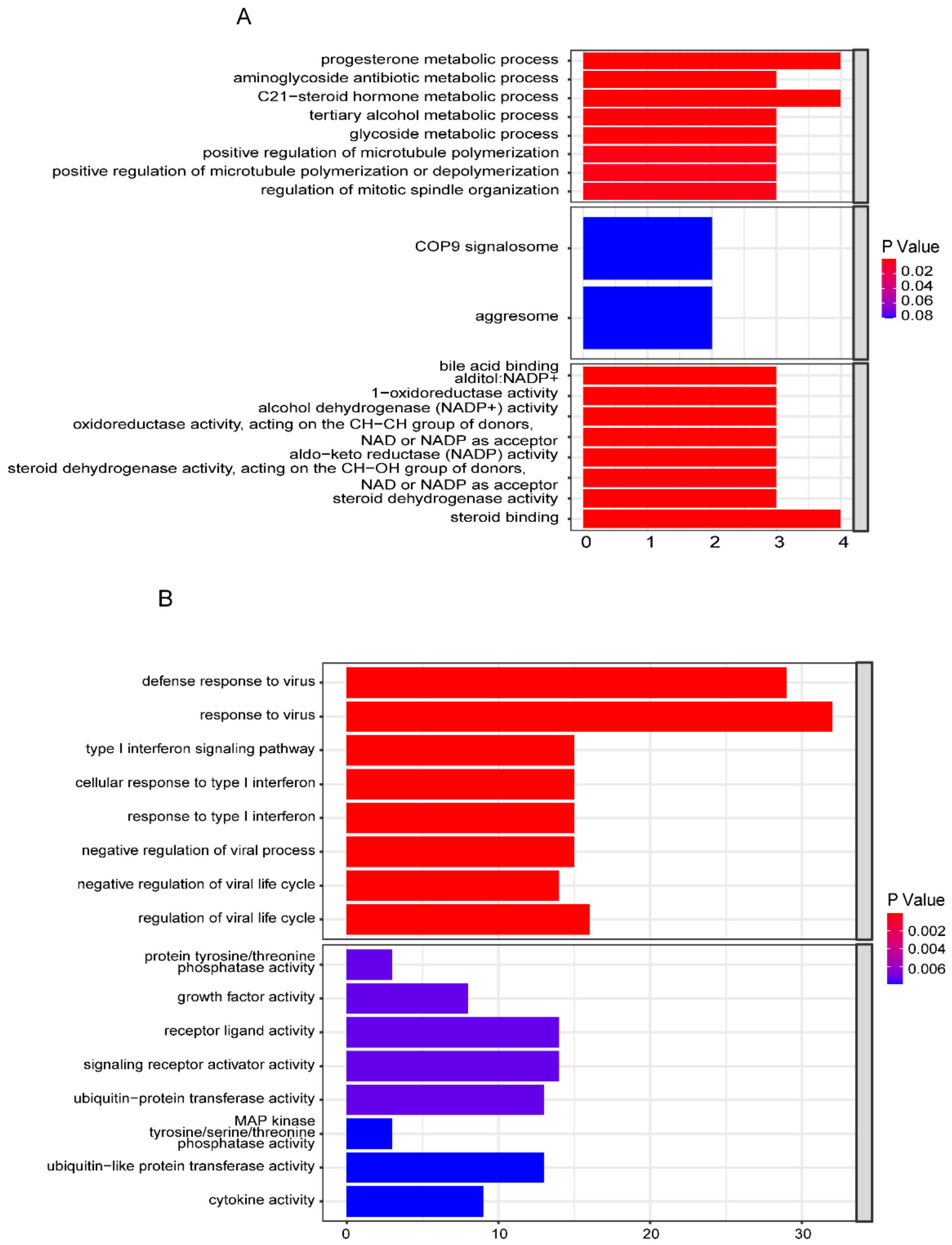

Figure S3. Analysis of HepG2 BLM and Control cells gene expression.

(A) Gene Oncology of the downregulated gene function. **(B)** Gene Oncology of the upregulated gene function.

Table S1. Antibodies information.

Antibodies	Source	Identifier
H3	Abclonal	AB_2631273 WB: 1:10000
GAPDH	Abclonal	AB_2769670 WB: 1:5000
HDAC1	Abclonal	AB_2757051 WB: 1:5000, CUT&Tag: 1:200
H3K27ac	Abcam	AB_2828007 WB: 1:5000, IF: 1:200, CUT&Tag: 1:200
H3K27me3	Abcam	AB_2650559 WB: 1:5000, IF: 1:200, CUT&Tag: 1:200
H3R26me2a	Abclonal	AB_2764947 WB: 1:2000, IF: 1:100, CUT&Tag: 1:50
H3R26me2s	Abclonal	AB_2769670 WB: 1:1000, IF: 1:100, CUT&Tag: 1:50
Lamin B1	Abclonal	AB_2861583 WB: 1:5000
γH2A.X	Abclonal	AB_2771168 WB: 1:2000, IF: 1:100, CUT&Tag: 1:100
Flag-Tag	Abclonal	AB_2770401 WB: 1:8000
PRMT5	Abclonal	AB_2762092 WB: 1:5000

Table S2. Cell lines information.

Notes: the Cell Bank of the Chinese Academy of Science is not included in RRID.

Cell lines	Source	Catalog	Identifier
HepG2	Cell bank, Chinese Academy of Sciences.	SCSP-510	CSTR:19375.09.3101HUMSCSP510
LM3	Cell bank, Chinese Academy of Sciences.	SCSP-5093	CSTR:19375.09.3101HUMSCSP5093
A549	Cell bank, Chinese Academy of Sciences.	SCSP-503	CSTR:19375.09.3101HUMSCSP503
Hela	Cell bank, Chinese Academy of Sciences.	SCSP-504	CSTR:19375.09.3101HUMSCSP504
293	Cell bank, Chinese Academy of Sciences.	SCSP-5209	CSTR:19375.09.3101HUMSCSP5209
HFF	Cell bank, Chinese Academy of Sciences.	SCSP-109	CSTR:19375.09.3101HUMSCSP109
